# GLI1 finds a new role in cancer stem cell biology

**DOI:** 10.1002/emmm.201302505

**Published:** 2013-03-18

**Authors:** Martin E Fernandez-Zapico

**Affiliations:** Division of Oncology Research, Schulze Center for Novel Therapeutics, Mayo ClinicRochester, MN, USA

**Keywords:** GLI1, NRP2, therapy, triple negative breast tumors, tumor initiating cells

Tumor initiating cells (also known as cancer stem cells) are a subset of cells with the ability to self-renew and differentiate into the heterogeneous populations of the original tumour (Magee et al, 2012; Sampieri & Fodde, 2012; Zhou et al, 2009). This constitutes the cancer stem cell hypothesis, which is gaining significant attention because it may explain resistance to therapy and tumour recurrence (Zhou et al, 2009). Tumour initiating cells were first described in leukaemias in the early 1990s and more recently in an increasing number of solid tumours (Bonnet et al, 1990), especially the poorly differentiated ones (Sampieri & Fodde, 2012). Tumour initiating cells can originate from the transformation of normal tissue stem cells or derive from an existing cancer cell population. In certain tumours, this cell population is present in specific niches that make them more resistant to treatment to therapy (Magee et al, 2012; Zhou et al, 2009). Although much work is still needed to identify and characterize the biology of tumour initiating cells, efforts are now being directed towards designing therapeutic strategies to target this cellular compartment. The characterization of the specific signalling pathways controlling the biology of these cells is thus needed to help design future clinical studies to overcome the limitations of current therapies against neoplastic diseases.

**Figure 1:**
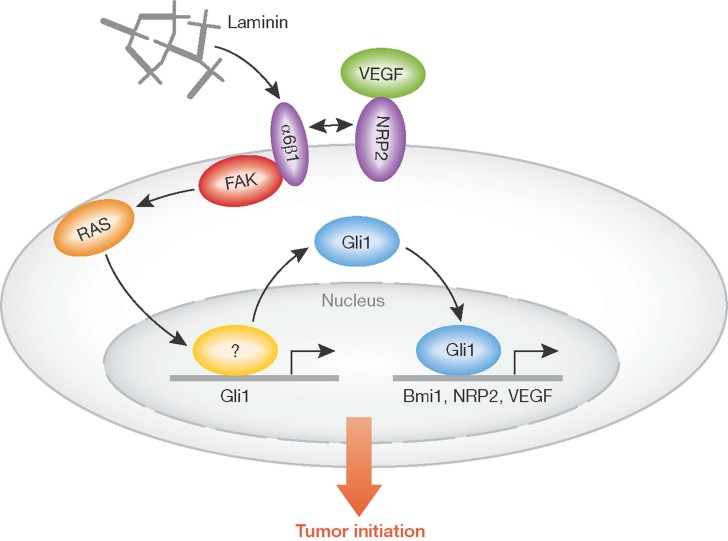
Schematic representation of the novel NRP2-α6β1-FAK-RAS-GLI1 axis involved in the regulation of tumour initiating cells in breast cancer. The pathway is activated by NRP2 and α6β1 integrin, which in turn induces FAK/Ras pathway leading to the activation of GLI1 and the consequent increase in NRP2 and BMI-1 expression.

In this issue, a study by Goel and colleagues defines a novel pathway regulating the biology of tumour initiating cells in triple negative breast cancers (Goel et al, 2013) (Fig 1). These tumours are defined by their lack of expression of the estrogen receptor α, progesterone receptor and HER2, and are generally of high histological grade, poorly differentiated and more aggressive compared to other subtypes of breast cancer (Irshad et al, 2011). Triple negative breast cancers are highly resistant to therapy and long-term favourable outcomes are rare. Hence, there is an urgent need to improve current therapeutic regimens for these patients. It is thought that early recurrence is in part due to the fact that tumour initiating cells are resistant to conventional therapies, can remain dormant for extended periods and can subsequently give rise to secondary tumours. In addition, data from preclinical models and clinical trials demonstrate that chemotherapy and radiation can induce and select for tumour initiating cells. New therapeutic treatments for triple negative breast cancers should be developed to overcome this problem. Therefore, identification of pathways generating and maintaining tumour initiating cells is of key importance to help achieve this goal.

Goel et al. have identified a novel cascade downstream of the VEGF receptor Neuropilin-2 (NRP2) regulating the biology of tumour initiating cells (Fig 1). NRP2, a neuronal receptor involved in axon guidance (Uniewicz & Fernig, 2008), is highly expressed in human breast cancer and correlates with aggressive disease and poor clinical outcome, features of triple negative breast cancer. NRP2 maintains the tumour initiating cell compartment by stimulating α6β1 integrin, FAK-mediated activation of Ras signalling and consequent induction of the zinc finger transcription factor and Hedgehog effector GLI1, which in turn induces the stem cell factor BMI1 (Lobo et al, 2007), enhances NRP2 expression and α6β1 function, thus establishing a positive autocrine loop. Further characterization of the mechanism identified NRP2 and BMI1 as direct targets of GLI1, which binds to the promoter of these molecules to regulate their expression. *In vivo* experiments using transgenic mice overexpressing GLI1 in the mammary gland show an increase in the tumour initiation cell population, thus further supporting a role of this molecule in the regulation of this cellular compartment. Finally, using an antibody-based inhibition strategy, the authors show that this pathway can be effectively targeted in a triple negative breast cancer model thus reducing tumour burden and recurrence.

Goel et al. have identified a novel cascade downstream of the VEGF receptor Neuropilin-2 (NRP2) regulating the biology of tumour initiating cells.

Collectively, these findings define several novel mechanistic aspects of the biology of tumour initiating cells as well as provide the foundation for new therapeutic approaches targeting this cellular compartment. However, the study also defines new gaps in our knowledge as well as future directions that investigators in this field of study should consider. First, at the molecular level, the discovery of the zinc finger transcription factor GLI1 as a central player in the regulation of stemness should fuel future efforts investigating the epigenetic mechanisms underlying the regulation of tumour initiating cells, especially their role in cell differentiation, a key cellular process in the biology of these cells. In contrast to genetics, epigenetic processes are highly dynamic, reversible (McCleary-Wheeler et al, 2012) and define the response of a cellular compartment (*e.g.* tumour initiating cells) to defined stimuli (*e.g.* chemotherapy). Increased understanding of the mechanisms maintaining cancer cell stemness and controlling the differentiation of tumour initiating cells will be important for future translational efforts. In particular, the molecular mechanisms regulating chromatin and DNA-based epigenetics are clinically relevant because drugs targeting different complexes regulating these events are currently undergoing clinical trials (*e.g.* histone deacetylase or methyl transferase inhibitors; Lee et al, 2012; Zagni et al, 2012). At the cellular level, the fact that several components of this newly identified loop have been reported to drive GLI1 transcriptional activity independently of the VEGF-NRP2 pathway in cancer cells suggests the presence of signalling pathways that could overcome anti-NRP2 therapies by keeping an active GLI1 downstream of this receptor (Hui & Angers, 2011; Lauth & Toftgård, 2011; Perrot et al, 2013; Aberger et al, 2012; Ji et al, 2007; Mangelberger et al, 2012). In addition, the presence of this alternative signalling pathway regulating GLI1 supports the existence of different tumour initiating cell types. Hence, subtyping of these cells will be of importance in better predicting benefit from novel targeted drugs. Due consideration should also be given to the tumour microenvironment, which expresses multiple factors with the ability to modulate GLI1 activity (Hui & Angers, 2011; Lauth & Toftgård, 2011; Mangelberger et al, 2012). Defining the impact of the tumour microenvironment on this molecular process and identifying the entire repertoire of signalling pathways activating GLI1 will be crucial for the success of future anti-NRP2 therapies. Finally, from a translational point of view, the study provides the rationale for future clinical trials, although establishment of the best combination therapy as well as regimen design will arise from additional preclinical studies in relevant cancer stem cell models. Efforts in the field should be directed at the generation of new animal models recapitulating the features of tumour initiating cells and their interaction with other cellular compartments within the tumour microenvironment.

…the discovery of the zinc finger transcription factor GLI1 as a central player in the regulation of stemness should fuel future efforts investigating the epigenetic mechanisms underlying the regulation of tumour initiating cells
